# Personality as a Factor Underlying Cognitive Reserve

**DOI:** 10.1002/alz.086450

**Published:** 2025-01-09

**Authors:** Annabell Coors, Seonjoo Lee, Christian G Habeck, Yaakov Stern

**Affiliations:** ^1^ Cognitive Neuroscience Division, Columbia University Vagelos College of Physicians and Surgeons, New York, NY USA; ^2^ Taub Institute for Research in Alzheimer’s Disease and the Aging Brain, Columbia University, New York, NY USA; ^3^ Columbia University Irving Medical Center, New York, NY USA; ^4^ Columbia University, New York, NY USA; ^5^ Cognitive Neuroscience Division, Columbia University, New York, NY USA

## Abstract

**Background:**

Cognitive reserve is a concept that explains why cognitive performance can be better than would be expected given the level of brain pathology. In this project, we investigated whether certain personality traits are a factor underlying cognitive reserve. If so, individuals with these personality traits would show better cognition and less cognitive decline than would be expected given their level of age‐related brain changes.

**Method:**

The cross‐sectional analyses were based on 399 healthy adults aged 19‐80 and the longitudinal analyses were based on 273 individuals. The mean follow‐up time was 5 years (SD = 0.7 years). The International Personality Item Pool questionnaire was used to assess the BIG5 personality traits openness, conscientiousness, extraversion, agreeableness, and neuroticism. Each cognitive domain (perceptual speed, memory, fluid reasoning, and vocabulary) was measured with up to 6 tasks. Cognitive domain‐specific brain status variables, derived from 77 structural brain measures using elastic net regularization, explained up to 43.1% of variance in cognitive performance.

**Result:**

Higher openness was associated with higher fluid reasoning and better vocabulary after adjusting for the cognitive domain‐specific brain status variables, age, and sex. Additionally, lower brain status was only associated with increased decline in perceptual speed among individuals with low openness. In contrast, in individuals with high openness, brain status was almost unrelated to change in perceptual speed.

**Conclusion:**

High openness increases cognitive reserve and should be promoted across the lifespan.

